# Supplementary material for: Are carbon emissions trading and green financial instruments synergistic? -Comprehensive quantitative research based on content analysis
Source: PLoS One. 2024 Mar 7;19(3):e0298601. doi: 10.1371/journal.pone.0298601 (PMC10919601; doi:10.1371/journal.pone.0298601)
Supplement: S1 Data — (ZIP) [file pone.0298601.s001.zip › Data/地级市Z_F工作报告.zip!/╜¡╦╒/╠⌐╓▌╩╨/└┤╫╘╣1⁄2╓┌║┼í╛┬φ┐╦╩2╛▌═°í┐_2016.docx]

各位代表：

　　现在，我代表市人民政府向大会作工作报告，请予审议，并请市政协委员和其他列席人员提出意见。

一、 2015年工作和“十二五”发展回顾

　　过去的一年，我们在中共泰州市委的正确领导下，坚持以党的十八大、十八届三中四中五中全会和习近平总书记系列重要讲话精神为指导，深入推进三大主题工作，加强多重目标、多种政策、多项改革的协调配合，统筹稳增长、调结构、惠民生、防风险，较好地完成了市四届人大四次会议确定的各项目标任务。

　　经济运行稳中有进。主动适应经济发展新常态，妥善应对下行压力，有效稳住了经济基本面。主要指标平稳增长。预计实现地区生产总值3620亿元，增长10%。一般公共预算收入322.2亿元，增长13.9%。固定资产投资2695.7亿元，增长22.6%。社会消费品零售总额突破1000亿元，增长10.9%。城乡居民人均可支配收入分别达34167元、16508元，分别增长9%、9.5%。居民消费价格涨幅1.7%。质量效益不断提升。规模以上工业实现增加值2465.4亿元，增长11%；主营业务收入10700亿元、利税1350亿元、利润800亿元，分别增长14.8%、19.5%、18.5%。新增销售10亿元以上企业8家、利税过亿元企业19家。单位地区生产总值能耗下降5%，主要污染物排放量削减完成年度目标任务。运行质态持续向好。本外币各项贷款余额突破3300亿元，新增人民币贷款476.6亿元。新增主板上市企业1家、“新三板”挂牌企业14家，净增直接融资154.6亿元。客货运周转量增长11.7%，港口吞吐量增长6.2%。新增市场主体4.3万户、企业注册资本816.7亿元，分别增长6.6%、55.4%。

　　转型升级步伐加快。推进经济结构战略性调整，制定实施工业十条、开放十条、人才十条、金融十条，在加快转型升级中厚植发展优势。有效投资不断扩大。把项目大突破的重点放在产业项目上，新开工亿元以上项目331个、在建436个，均比上年增长近一倍。完成产业投资2218亿元，增长36.4%，其中工业投资1635亿元，增长36.6%，增幅均居全省首位。鼓励支持企业加强技术改造，完成工业技改投入882亿元。产业结构进一步优化。大力发展高效农业、设施农业，新增家庭农场1500多家、省级农业产业化龙头企业7家，综合机械化率超过80%。市农业开发区获批国家农业科技园区，苏台（姜堰）农业合作创业园获批省级园区。培育壮大战略性新兴产业，布局发展大健康产业，实现新兴产业产值2550亿元。推进“两化”深度融合，改造提升传统产业，装备制造业产值突破6000亿元，造船完工量占全国的15.2%，我市成为全国首家两化融合管理体系贯标试点城市。推动“建筑强市”建设，实现产值2950亿元，增长14.4%，2项承建工程获评“鲁班奖”。加快发展服务业，实现增加值1614亿元，增长10.5%，省级现代服务业集聚区达9家，兴化、高港获批省级农村电子商务示范县。全年接待游客2089万人次，实现旅游总收入250亿元，秋雪湖生态景区获批国家4A级景区。科技创新成效突出。研发经费支出占地区生产总值比重达2.35%。高新技术产业实现产值4752亿元，增长16%。新认定高新技术企业137家，新增省高新技术产品789个。建成“泰科易”网上技术交易平台，获批省产学研前瞻性联合研究项目3个，新获发明专利授权550件。引进高层次人才2157名，新增国家“千人计划”专家11名、省“双创团队”3个。扬子江药业获评全国质量标杆企业，全市新增中国驰名商标11件。

　　改革开放不断深化。以经济体制改革为主轴，实施全面深化改革两年行动计划，重点改革推进有力。坚持以改革转换发展动力、集聚发展优势、创造发展红利，创新券、创业券、购房券“三券联动”走在全省前列。率先实施企业名称自主核准、注册登记全程电子化等商事制度改革，“三证合一”、“一照一码”实现市区全覆盖。“六个一”试点扎实推进，医药高新区行政审批基本实现“园内事园内结”。深化财税、金融、投融资改革，引入第三方开展财政资金绩效评价，设立中小企业转贷资金，获准筹建苏州银行、平安银行泰州分行，组建市凤城国资控股有限公司。机关事业单位养老保险制度改革稳步推进，公务用车改革基本完成。抓住国家新一轮扩大开放机遇，主动融入“一带一路”、长江经济带建设等重大战略，开放开发水平进一步提升。精心组织北上广深投资促进周、泰台经贸交流合作促进月等招商活动，全力招引战略型、龙头型、配套型项目，全年新签亿元以上项目583个，实际利用外资10.65亿美元，增长13.4%。外经外贸平稳发展，完成进出口总额101亿美元，实现外经营业额8.5亿美元，获批省跨境电子商务试点城市。加快中国医药城产业化步伐，瑞士雀巢、法国高德美等192个医药类项目签约落户，迈博太科、中崇信诺等51个项目开工建设，阿斯利康三期、海王保健品等39个项目竣工投产。持续推进特色园区建设，泰兴经济开发区创成国家知识产权试点园区，市数据产业园、海陵工业园创成省级电子商务示范基地。放大园区南北共建效应，锡山经济开发区兴化工业园、江阴高新区黄桥工业园和昆山高新区姜堰工业园累计引进项目投资超100亿元。海关、国检、海事、边检为开放开发作出积极贡献。

　　城乡建设扎实推进。中心城市建设速度加快。西客站片区、西南城河景观带等老城改造项目进展顺利，稻河古街区建成开街。现代服务业聚集区（金融服务区）、省泰中新校区、市人民医院新区医院等新城重点工程取得积极进展，周山河生态环境工程启动实施。东风路、永定路快速化改造有序推进，东环高架路建成通车。城市环境综合整治项目全面完成，济川路立面改造与亮化工程顺利竣工。重大基础设施建设统筹推进。新一轮港口建设各项前期工作全面展开，泰镇高速、阜兴泰高速泰州段和京沪高速江广段扩容完成序时任务，宁启铁路复线电气化改造全面完工，泰东河、川东港整治等重点水利工程加快实施。新型城镇化建设力度加大。加快培育小城市，落实流动人口“同城待遇”，城镇化率提高到61.6%。办好新一轮农村实事，乡镇管道燃气覆盖率达80%，镇村公交开通率达67%，新改建农村公路338公里，新整治县乡村河道1002条。生态环境建设取得突破。国家生态市创建达到考核标准，全国城市环境总体规划编制试点、水生态文明城市建设试点工作有力有序，高港创成省可持续发展试验区，兴化戴南科技园区获批国家“城市矿产”示范基地。加强大气、水、土壤污染防治，完成城市黑臭河道整治年度任务，空气质量优良率提高5.4个百分点。全市新增造林面积4.2万亩，市区新增绿地面积300万平方米，完成“空转绿”工程项目53个。

　　人民生活持续改善。强化就业和保障工作。落实全民创业30条和大学生创业引领计划，新增城镇就业10万人、创业6.2万人，城镇登记失业率1.89%。城乡基本养老、基本医疗和失业保险扩面提标，城乡低保标准、城镇居民医保和新农合补助标准进一步提高，社会救助和残疾人服务体系不断完善。新建城乡社区居家养老服务中心（站）416家。大力发展社会事业。教育现代化建设扎实推进，学前教育省优质园比例达70%，义务教育全域均衡发展，高中教育质量稳步提升，泰职院成为省级示范性高职院校立项建设单位，泰州学院师资队伍建设、科研项目申报实现突破。城市公立医院综合改革等医改工作有序推进，国家卫生城市复审顺利通过。“百姓”系列、文化惠民活动深入开展，梅兰芳艺术节、微电影创作等品牌影响力不断扩大。创成省公共体育服务体系示范区，成功举办全国业余铁人三项赛等重要赛事。民族宗教、史志档案、社会科学、广播电视、妇女儿童工作取得新进步，统计、气象、防震、民防、外事侨务、对台工作取得新成绩。切实维护社会稳定。推进城乡社区网格服务管理规范化建设，落实领导干部接访下访、社会稳定风险评估制度，有效预防和化解社会矛盾。加强社会治安综合治理，打击各类违法犯罪活动，建成四级综治信息系统专网。推进法律援助和普法工作，通过“六五”普法终期考核。强化安全生产监管，安全事故起数、死亡人数实现双下降。开展食品药品安全专项整治，保障人民群众饮食用药安全。做好国防动员和双拥优抚安置工作，备战训练质量全省领先。

　　自身建设得到加强。坚持依法行政。落实行政执法责任制，严格规范性文件制发程序，全面建立政府法律顾问制度，行政负责人出庭应诉率达99.1%。主动接受人大的法律监督、工作监督和政协的民主监督，广泛听取各民主党派、工商联、无党派人士和社会各界意见，认真办理人大代表建议和政协提案，办成率超过70%。深化政务公开，推广电子政务和网上办事。坚持改进作风。组织开展“三严三实”专题教育，认真贯彻中央八项规定和省、市委十项规定精神，坚持不懈纠正“四风”。定期组织企业家座谈，深入开展“三服务”活动，建立健全工业企业服务联系制度，着力营造“新三比”服务环境。严格执行民生工作例会制度，充分发挥12345·政风行风热线、市长信箱和望海楼论坛等平台作用，协调解决群众反映强烈的热点难点问题。坚持勤政廉政。全面落实党风廉政建设责任制，加强重点领域廉政风险防控，严肃查处腐败案件和不正之风。强化行政监察和审计监督，严格监管公共资金、国有资产、国有资源和领导干部履行经济责任情况，审计整改问责机制逐步完善。践行“马上就办、真抓实干”，开展“四比四查”专项行动，机关效能进一步提升。

　　我们也清醒地认识到，工作中还存在一些问题和不足。主要是：经济实力和综合竞争力还不够强，又好又快发展的任务仍然很重；经济下行压力不断加大，部分行业企业经营困难，一些领域潜在风险不容忽视；中心城市功能仍不完善，新型城镇化建设有待加快；城乡居民持续增收难度加大，改善民生需要付出长期努力；政府职能转变还不到位，领导经济社会发展的能力和水平亟待进一步提升，等等。这些问题，我们一定高度重视，采取有效措施，认真加以解决。

　　各位代表，刚刚过去的2015年是“十二五”规划的最后一年。五年来，我们积极应对国内外宏观环境变化，谋划落实改革创新、追赶跨越的各项举措，致力走好转型发展、融合发展的泰州之路，全市经济社会发展取得重大成就。

　　这五年，我们围绕转变发展方式，始终紧扣转型升级主线。把稳定经济增长作为重中之重，把推动转型升级作为根本出路，努力实现经济发展调速不减势、量增质更优。综合实力实现跃升。2015年地区生产总值是“十一五”期末的1.8倍，年均增长11.4%。规模以上工业总产值、主营业务收入均突破1万亿元，增加值较“十一五”期末翻了一番。一般公共预算收入突破300亿元，年均增长13.5%。累计完成固定资产投资9312亿元，是“十一五”的2.5倍。产业质态加快提升。实施传统产业升级、新兴产业双倍增计划，装备制造业加快向产业链高端攀升，生物医药产销规模全省领先，电子信息、新能源、新材料等产业加速成长。实施服务业提速计划，增加值占比年均提高1.4个百分点。实施农业现代化工程，国家现代农业示范区建设达到基本现代化水平。三次产业结构由2010年的7.4︰55︰37.6调整为6.0︰49.4︰44.6。实体经济支撑有力。实施千企升级百企示范工程，累计净增规模以上企业440家，新增销售50亿元、利税10亿元以上企业分别达24家、18家，新认定高新技术企业424家。

　　这五年，我们围绕增强内生动力，深入实施双轮驱动战略。持续推动开放创新“两个轮子”一起转、协调转，在开放中高起点创新，在创新中全方位开放，培育发展动能，拓展发展空间。开放层次进一步提高。中国医药城建成国家新型工业化产业示范基地，泰州综合保税区成功获批，靖江经济开发区升格为国家级开发区。泰州大桥通车，扬泰机场通航，泰州港迈入亿吨大港行列，核心港区5万吨级集装箱码头开港运营。累计实际利用外资61.9亿美元，新落户世界500强企业14家，进出口总额是“十一五”的1.9倍，外经工作跻身全省第一方阵。创新能力进一步增强。健全完善区域创新体系，连续五次入选国家科技进步先进市，获批国家创新型试点城市和国家知识产权示范城市，高新技术产业产值占比较2010年提高9.8个百分点。累计新建产学研联合体710家、省级工程技术研究中心66家，专利授权量是“十一五”的4倍。注重强化人才支撑，累计引进高层次人才8211名，全市拥有国家“千人计划”专家39名。

　　这五年，我们围绕打造特色优势，着力推进三大主题工作。坚持从问题导向、发展导向入手，逐步确立新的发展支点和工作抓手，全力以赴推进思想再解放、项目大突破、城建新提升，致力构建区域竞争新优势。深化改革取得新进展。把思想解放的成效落实到改革攻坚上，深入推进行政管理体制、资源要素市场化、科技创新体制、绿色发展机制、区域统筹发展机制等重要领域和关键环节改革，转型升级综合改革试点工作初见成效。项目建设形成新高度。着力完善项目招引、推进和考核体系，深入实施转型升级“双百”工程，项目总量、体量、质量均实现历史性突破，累计完成产业投资是“十一五”的2.1倍。总投资86亿元的国电二期正式投产，102亿元的中海油一体化即将竣工，60亿元的环氧丙烷及配套项目开工建设，30亿元的汇福粮油、28亿元的高效单晶电池、7亿美元的欣兴电子等项目签约落户。中心城市建设步入新阶段。姜堰顺利撤市设区，城市发展空间和能级明显提升。组织开展第四轮城市总体规划修编，调整完善市区城建体制和房屋征收政策。启动实施城建新提升两年行动计划，老城改造、新城建设、交通优化、水环境整治、景观绿化等重点工程扎实推进。创成全国文明城市，获批国家历史文化名城。

　　这五年，我们围绕增进民生福祉，加快构建七大公共服务体系。牢固树立“发展经济、造福百姓”理念，大力实施民生幸福工程，让发展成果更多惠及全体人民。注重多办实事、务求实效。确保每年一般公共预算支出的70%、新增财力的80%以上用于改善民生，集中力量办成了一批民生实事。落实“十项富民行动计划”，千方百计增加城乡居民收入。改善农村生产生活条件，区域供水通水乡镇、污水处理设施实现建制镇全覆盖，村庄环境整治通过省级验收并获省人居环境范例奖。注重夯实基础、优化服务。坚持以创业带动就业，获评全国创业先进城市。各市（区）均通过区域教育现代化省级评估，全部建成国家义务教育发展基本均衡市（区）。社会保险制度实现全覆盖，主要险种覆盖率稳定在97%以上。医药卫生体制改革深入推进，县级公立医院全部创成三级医院。优先发展公共交通，公交出行分担率逐年提高，公共自行车服务系统建成投用。加强保障性住房建设，住房保障体系健全率达82.5%。扶持发展养老服务业，每千名老年人拥有养老床位38张。注重创新治理、促进和谐。刑事案件发案率连续5年全省最低，连续4年获评全省综治平安建设先进市，群众安全感保持全省前列。

　　“十二五”经济社会发展取得的成就，是全市人民在中共泰州市委领导下，齐心协力、开拓进取、扎实工作的结果，与市人大、市政协的监督支持密不可分。在此，我代表市人民政府，向在各条战线上付出辛勤劳动的广大干部群众，向给予政府工作热情支持和有力监督的全体人大代表、政协委员，向各民主党派、工商联、无党派人士、各人民团体和广大离退休老同志，向中央和省驻泰机构以及驻泰部队指战员、武警官兵和政法干警，向所有关心支持泰州发展的海内外朋友们，表示衷心的感谢！

二、“十三五”发展目标任务

　　各位代表，“十三五”是我市全面建成小康社会的决胜时期，也是提升泰州在全省乃至长三角、长江经济带城市地位的关键阶段。为谋划未来五年发展，2014年以来，市政府组织开展了“十三五”规划编制工作。党的十八届五中全会以后，按照中央和省委的要求，根据市委四届九次全会通过的《中共泰州市委关于制定泰州市国民经济和社会发展第十三个五年规划的建议》，市政府制定了《泰州市国民经济和社会发展第十三个五年规划纲要（草案）》，提请大会审议。

　　“十三五”时期，经济社会发展指导思想是：全面贯彻党的十八大、十八届三中四中五中全会精神，以马克思列宁主义、毛泽东思想、邓小平理论、“三个代表”重要思想、科学发展观为指导，深入贯彻习近平总书记系列重要讲话精神特别是视察江苏重要讲话精神，坚持“四个全面”的战略布局，紧盯率先全面建成小康社会的核心任务，围绕“经济强、百姓富、环境美、社会文明程度高”的目标定位，坚持发展是第一要务，以提高发展质量和效益为中心，以三大主题工作为抓手，深入实施“八项工程”，全面推进创新发展、协调发展、绿色发展、开放发展、共享发展，加快形成引领经济发展新常态的体制机制和发展方式，着力建设以大健康产业集聚为特色的中国医药名城，以江海联动、工贸发达为特色的长江经济带港口名城，以文昌水秀、古今交融为特色的国家历史文化名城，以水城水乡、都市花园为特色的长三角地区生态名城，打造人民群众有更多自豪感、幸福感、归属感的“康泰之州、富泰之州、祥泰之州”，谱写好中华民族伟大复兴中国梦的泰州篇章。

　　“十三五”时期，经济社会发展主要目标是：地区生产总值年均增长8%以上，到2020年达5500亿元左右，提前实现比2010年翻一番。一般公共预算收入、城乡居民收入实现与经济基本同步增长。固定资产投资年均增长15%左右。社会消费品零售总额年均增长10%左右。节能减排完成省定目标任务，环境质量达到省定现代化标准。

　　实现“十三五”发展目标，必须牢固树立并自觉践行五大发展理念，全面落实结构性改革新要求，主动适应和积极引领经济发展新常态，坚定不移地把发展作为第一要务，坚定不移地推进三大主题工作，坚定不移地致力稳中求进、稳中奋进，认真落实五个方面重点任务：一是坚持创新发展，着力构建特色鲜明、高端引领的产业结构。进一步聚焦生物医药及高性能医疗器械、高技术船舶及海工装备、节能与新能源产业发展，力争形成5000亿元规模；落实《中国制造2025泰州实施纲要》，推动传统产业向中高端迈进；提速提质发展生产性、生活性服务业，加快形成以服务经济为主体的经济结构；转变农业发展方式，确保农业现代化水平全省领先。发挥科技创新的引领作用，积极发展新产业、新技术、新业态、新模式，科技进步贡献率达61%以上，建成创新型城市。强化消费、投资、出口的协调拉动，完成全面深化改革各项任务，构筑经济发展的新动能。二是坚持协调发展，着力构建统筹互动、融合并进的城乡结构。以构建“一大三中九小”新型城镇体系为重点，推动中心城市能级提升，推进县域城市规划建设，加快培育特色小城市。以融入长三角核心区、优化市域生产力布局为重点，创新区域融合机制，促进生产要素流动、资源优化整合。以同步推进物质文明和精神文明建设为重点，深化全国文明城市创建，加快文化产业和事业发展，丰富新型城镇化和城乡一体化内涵。三是坚持绿色发展，着力构建人与自然和谐发展的生态结构。围绕创建国家生态文明建设示范区，实施主体功能区战略，推行绿色生产模式，完善生态保护机制，促进绿色产业发展；突出环境治理和生态建设并举，落实“四大行动计划”，促进绿色环境建设；牢固树立绿色政绩观，大力弘扬生态价值观，促进绿色风尚形成。四是坚持开放发展，着力构建层次更高、融入更深的开放结构。把要素集聚作为首要任务，持续推进招商引资、项目突破和开发园区提档升级。力争累计利用外资100亿美元，完成产业投资1.5万亿元，培植1000亿级、500亿级园区分别达3家、8家以上。把港口建设作为开放龙头，加快完善集疏运体系，全力打造江海联运中心港，推动以港聚产、以产兴城、港产城一体化发展。把交通优化作为关键支撑，完善公铁水空立体交通网络，提升现代综合交通运输效率。五是坚持共享发展，着力构建普惠均衡、文明和谐的社会事业结构。立足实现全领域、全人口、全地域的全面小康，落实城乡居民收入倍增计划，推进新一轮脱贫帮扶工作，加快共同富裕步伐；实施七大公共服务体系提升工程，增强公共产品供给能力，创新公共服务提供方式，提高共建共享水平；深化平安泰州建设，健全公共安全体系，全面推进依法治市，打造全省平安建设示范区、法治建设先导区、社会治理样板区。

三、 2016年工作任务

　　今年是建市20周年，是实施“十三五”规划的开局之年，也是展示三大主题工作阶段性成果的关键之年。我们将认真落实中央和省经济工作会议、省委十二届十一次全会、市委四届九次十次全会决策部署，深入推进三大主题工作，切实加快“四个名城”建设，着力加强供给侧结构性改革，在“去产能、去库存、去杠杆、降成本、补短板”上打好组合拳，全力抓好“五大任务三十条举措”的落地见效，努力实现“十三五”经济社会发展的良好开局。

　　全市国民经济和社会发展主要预期目标为：地区生产总值增长9%以上，一般公共预算收入、城乡居民收入实现与经济基本同步增长，固定资产投资增长16%左右，社会消费品零售总额增长10%左右，居民消费价格涨幅3%左右，城镇登记失业率控制在3.5%以内，研发经费支出占地区生产总值比重2.4%左右，节能减排完成省定目标任务。

　　今年将重点抓好八个方面工作：

　　1. 在转型升级中保持经济平稳健康发展。坚持调高调轻调优调强基本取向，以增量优化培植后劲，以存量调整提高效率，不断增强产业和企业竞争力。

　　持续推进项目建设。把好产业项目准入门槛，健全投资项目审核联动机制，从源头上杜绝产能过剩行业新上项目。围绕我市主导产业，策划一批具有市场前景、符合产业方向的特色项目，储备并实施一批产业链高端环节、缺失环节项目，形成项目滚动发展格局。以转型升级“双百”工程为重点，严格执行督查推进机制和挂钩联系制度，加快436个在建亿元以上项目建设，推动中海油一体化、扬子江龙凤堂中药产业基地、纬创资通显示器等项目尽快投产达效。全年力争新开工亿元以上产业项目315个，完成产业投资2500亿元。

　　务实推动产业升级。紧盯医药城发展“第一个十年目标”，推进国家一类新药投资基金正式运作、医药高新区整体上市、长江经济带大健康产业集聚示范区建设、国家药品进口口岸城市创建、与中科院大化所共建生物医药研究院等重点工作，加快产业集聚，壮大产业规模。大力发展高技术船舶、特种船舶和海工装备，提高智能化制造水平和行业配套能力。以新能源汽车、光伏发电组件、动力电池等为重点，培育壮大节能与新能源产业。推进3个创新中心和“中国云制造示范平台”建设，大力实施企业互联网化提升计划和技术装备升级计划，为推动产业迈向中高端提供引领和支撑。扎实做好去产能工作，制定冶金、纺织、化工等产能富余行业转型升级行动方案，开展过剩产能行业资产重组试点，加大“两高一低”落后产能淘汰力度。突出抓好金融服务、科技服务、现代物流等生产性服务业发展，培植一批制造业服务化示范企业。着力培育旅游、健康、信息等消费增长点，积极做好商品住宅去库存工作，有效释放刚性需求和改善性需求。

　　筑牢实体经济根基。开展降低实体经济企业成本行动，全面落实支持企业发展的各项政策，进一步优化企业发展环境，净增规模以上工业企业150家。引导企业瞄准行业标杆加强技术改造，提高产品技术、工艺装备、能效环保水平。支持企业实施质量品牌战略，构建产品质量标准化体系，加大商标、名牌申创力度，提高市场竞争力。鼓励企业完善法人治理结构，加快培养高素质本土企业家队伍。突出资产处置和人员安置两个重点，对资不抵债、扭亏无望的“僵尸企业”加快兼并重组或依法破产，为发展高端产业腾出资源和空间。切实做好去杠杆工作，防范和化解企业联保、非法集资等金融风险，坚决守住不发生系统性、区域性风险的底线。

　　2. 坚持以创新驱动提高发展质量和效益。推进以科技创新为核心的全面创新，着力强化“四个对接”，加快形成新的增长动力源泉。

　　强化科技创新支撑作用。实施高新技术企业“千企行动计划”，推进省级以上技术中心和创新示范企业建设，新增高新技术企业100家以上。探索产学研合作“离岸孵化”模式，加强产业技术创新联盟建设和关键核心技术联合攻关，高水平推进协同创新。开展发明专利攻坚行动，发挥好东大、南航、武汉理工等技术转移中心作用，实施一批前瞻性产业技术创新和重大科技成果转化专项。

　　推动大众创业万众创新。积极打造众创、众包、众扶、众筹支撑平台，落实众创空间实施方案，力争实现市（区）全覆盖。完善创业扶持政策体系和科技创业孵化链，鼓励支持大学生、企业高管等重点群体自主创业。大力发展民营经济，充分激发创新潜力，推动中小微企业和科技企业转型发展。

　　促进创新要素集聚整合。放大“三张劵”政策效应，实施高层次创新创业人才引进计划，深化科技成果产权制度和收益分配制度改革，更富成效集聚高端人才、技术和项目。加快科技广场建设，招引一批高端研发、成果转移转化、知识产权中介服务等机构。完善人力资源服务体系，实施“蓝领精英”培养工程，提高职业教育和技能培训水平，再造劳动力新优势。深入推进企业上市，积极发展科技金融，鼓励发展天使投资，力争成为国家级金融支持转型升级改革创新试点市。

　　3. 进一步拓展对外开放的广度和深度。主动融入国家开放大局，深入挖掘自身资源禀赋，着力塑造对外开放新优势。

　　提高开放型经济发展水平。推行精准招商，组织实施“招商引资266行动”，突出抓好先进制造业、现代服务业重大项目招引，力争实际利用外资实现较大幅度增长。壮大新兴出口主导产业，提高传统优势产品竞争力，加强服务外包示范区建设，推动产业优势转化为出口优势。引导企业抢抓“一带一路”建设机遇，加快“走出去”步伐，在境外投资建设贸易中心和生产加工基地。

　　推动开发园区提档升级。重点培育20个特色园区，鼓励发展“飞地经济”，促进产业集聚、企业集群。支持泰兴经济开发区和高港高新区创建国家级经济开发区，鼓励有条件的园区开展多形式合作共建。突出园区招商主阵地作用，强化招商考核，打造专业队伍，增强招商能力。抓好园区平台载体建设，优化要素资源配置，探索创新开发区体制机制和运营模式。

　　构建对外综合交通运输体系。加快与国际航运龙头企业的战略合作，推动组建港口投资运营实体，启动建设综合保税区B区。加大岸线和港口资源整合力度，谋划推进疏港铁路建设，促进港区运营、疏港交通一体化。推进连淮扬镇铁路淮泰联络线建设，做好盐泰锡常宜城际铁路、北沿江高铁前期工作，力争明年城际铁路开工建设。按期建成泰镇高速和京沪高速江广段扩容先导段，加快推进阜兴泰高速泰州段建设。完成通扬线三级航道建设年度任务。

　　4. 不断深化重点领域和关键环节改革。坚持“三个有利”标准，突出问题导向、精准发力、完善制度和督查落实，确保各项改革取得预期成效。

　　深化行政管理体制改革。继续推进行政审批制度改革，开展相对集中行政许可权和综合执法体制改革试点，进一步优化审批流程，提高审批效率。实施重点镇（园区）简政放权改革试点，做好省经济发达镇改革扩大试点工作。完善市场准入负面清单管理模式，强化事中事后监管。

　　深化市场配置要素改革。开展工业用地和三产用地调查，适时推行工业弹性用地制度，稳步实施中心城区建设用地利用评价和封闭挂钩试点。推进资源性产品价格改革，完善价格制定、监管、服务体系。进一步扩大排污权质押贷款试点，加快建设排污权交易二级市场，探索建立碳排放权、水权交易制度。

　　深化投融资体制改革。建立“借用还”相统一的举债融资机制，推进投融资平台实体化、市场化、公司化运行。清理整合闲置资金和专项资金，推行竞争性领域“财政扶持资金改基金”，探索组建市级城建基金、产业发展基金。开展政府与社会资本合作试点，积极上争国家专项金融债、建设债、建设基金等政策性金融产品。运用土地、价格、特许经营等政策杠杆撬动社会资本，探索形成公共服务和产品供给多元投入机制。

　　5. 加快提升中心城市功能品质。认真落实中央城市工作会议精神，提高城市规划建设管理水平，建设人民满意的幸福城市。

　　提升城市集聚能力。进一步完善城建体制，推动市区融合发展，启动东部片区改造工程，实施新328国道、姜高路、红旗大道东延和355省道西延，完成永定路城区段、东风路快速化改造和春兰路建设。推进老城改造，加快鼓楼北路和斜桥片区改造更新，完成西客站片区改造，提高稻河古街区、老街和三水湾运营水平。推进新城建设，扎实抓好现代服务业聚集区、市人民医院新区医院、医药城会展交易中心二期、泰职院新校区、体育公园等重大项目建设，配套完善基础设施和生活服务设施，按期建成省泰中新校区。推进以满足新市民需求为出发点的住房制度改革，发展住房租赁市场，改进拆迁安置方式，实现市区货币化安置全覆盖。

　　提升城市内涵魅力。深化历史文化名城保护，修缮税务街明清住宅、蒋科进士宅第等文保建筑，有序推进涵东、城中、渔行历史文化街区的原真性保护利用，加快建设盐税博物馆、评书评话博物馆等文化场馆。推进国家水生态文明城市建设试点，实施十大重点示范工程，形成人水和谐、水城交融的生态特色。全力打造周山河风光带，加快郊野公园规划设计和建设，完成重点片区“空转绿”工程。

　　提升城市管理水平。统筹地上与地下空间规划，推进地下综合管廊建设试点和海绵城市建设，启动实施稻河广场地下人防工程。开工建设餐厨废弃物处置中心，开展生活垃圾分类收集试点，更新提升环卫设施设备。加强城市管理数字化平台建设和功能整合，发展民生服务智慧应用，建设市区智能停车诱导系统。推进新一轮城市环境综合整治，进一步理顺城管行政执法体制。

　　6. 扎实抓好农业农村工作。坚持以新型城镇化推动城乡发展一体化，加快农业转方式、调结构、强基础，增强城乡发展的协调性。

　　大力发展现代农业。深入开展“互联网+现代农业”行动，着力发展绿色农业、循环农业、特色农业和品牌农业。推进国家现代农业示范区建设，重点打造城北现代农业走廊，提升农业园区发展水平。积极发展多种形式的适度规模经营，培育新型农业经营主体，新增市级示范家庭农场100家。实施农业重大技术推广计划，加强农机农艺配套，完善农业科技服务体系。以争创高标准农田建设资金整合试点市为抓手，推进农田水利基础设施建设，新增高标准农田15万亩。基本完成土地承包经营权确权登记颁证，开展供销合作社综合改革，巩固完善农村基本经济制度。

　　构建新型城镇体系。结合第四轮城市总体规划修编，完善城镇空间布局规划。加大小城市培育力度，强化重点镇产业和人口集聚功能，提高城镇建设的建成率、配套率、达标率，推进农民就地就近城镇化。创新城镇化投融资模式，吸引社会资本参与城镇建设。进一步优化村庄建设布局，引导农民向新型社区集中居住。坚持以“人的城镇化”为核心，深化户籍制度改革，促进有能力在城镇稳定就业和生活的农业转移人口进城落户。

　　加快城乡发展一体化。推进农业“接二连三”融合发展，优化整合乡镇工业集中区，积极发展农村电子商务、乡村旅游等新兴业态。深化全面小康村示范创建，实施新一轮农村实事工程。推进城乡基础设施共建共享，加快农村公路、电网改造升级，扩大供水供气覆盖面。推进城乡公共服务均等化，推动城乡低保、基本养老、医疗保险制度尽快并轨。

　　7. 持续加强生态环境建设。深入实施生态文明建设工程，加强优质生态产品供给，推动形成绿色生产方式和生活方式。

　　强化生态规划引领。编制完成主体功能区实施规划，调整扩大生态红线保护范围，合理控制开发边界和强度，落实生态补偿机制。加强资源环境承载能力监测预警，推进节约集约用地，抓好重点领域节能减排。严格执行项目能评和环评制度，推行企业清洁生产和园区生态化改造，启动建设泰州循环经济产业园。

　　加大污染防治力度。加强大气雾霾综合治理，严控建筑扬尘污染，深化秸秆综合利用和禁烧禁抛工作，有序推进充电站（桩）基础设施建设。继续实施城乡河道整治，加快污水处理厂和配套管网建设，完善安全供水保障体系。构建土壤环境监测和生物多样性保护网络，强化农业面源污染防治和工业污染场地治理。

　　完善生态保护机制。开展生态文明建设示范区创建，建立健全生态文明考核评价与责任追究机制，严格落实党政同责、一岗双责、损害生态环境终身追责等刚性要求。加大环保基础设施国有资本投入，推动社会资本建立环境保护基金，推行环境污染第三方治理。落实网格化监管措施，加强环保执法司法联动，严厉查处偷排直排等违法行为。

　　8. 切实保障和改善民生。把握坚守底线、突出重点、完善制度、引导预期的基本思路，实施七大公共服务体系提升工程，不断增强人民群众的获得感。

　　提高综合保障水平。大力推进富民工程，加强就业创业工作，持续增加城乡居民收入。聚焦黄桥老区、里下河经济薄弱地区和低收入农户，深入实施精准扶贫、精准脱贫，启动实施脱贫致富奔小康工程。健全社会保障体系，强化以五项社会保险为重点的基本保障，统筹衔接社会救助制度，发展社会福利与慈善事业。加强住房保障和供应体系建设，规范保障性住房管理机制。加快发展多层次社会化养老服务，探索居家养老服务新模式，建设标准化社区居家养老服务中心100家。

　　积极发展社会事业。促进义务教育优质均衡发展，大力发展普惠性学前教育，进一步提升职业教育、高中教育、高等教育发展水平，推动靖江、姜堰创成教育现代化建设示范区，着力构建“卓越教育体系”。启动“健康泰州”建设，深化公立医院改革，筑牢基层医疗卫生服务网底，切实增强基本医疗、基本公共卫生服务能力。落实“二孩”生育和计生困难家庭关爱政策，协调发展竞技体育和群众体育。探索设立“文化惠民券”，推进四级公共文化设施建设，抓好文艺精品创作生产，打造吉祥文化产业集群。落实文明城市创建长效机制，不断提升社会文明程度。

　　加强和创新社会治理。发挥社会治安综合治理功能，加强综治信息系统标准化建设，打造立体化防控体系。坚持重大决策社会稳定风险评估制度，健全多元化纠纷解决机制，依法规范信访秩序。深化民生领域法律服务，实现法律援助由低保人群向低收入人群延伸覆盖。推动民办非企业单位健康有序发展，推进“政社互动”和社区减负，增强基层自治活力。强化国防动员和后备力量建设，扎实做好优抚安置工作。加强安全生产风险隐患防控和系统治理，完善食品药品安全技术支撑体系，提高突发事件应急处置能力，全力维护社会和谐稳定。

四、 加强政府自身建设

　　站在两个五年规划的交汇点上，政府工作面临的形势严峻复杂，任务艰巨繁重。我们将坚持科学施政、依法行政、从严治政，与时俱进推进政府自身建设，努力开创政府工作新局面。

　　创新理念促发展，把握工作主动权。提升新常态下“有所为有所不为”的理性认识，遵循发展规律，践行“三严三实”，正确处理好经济效益、社会效益、生态效益的关系，全面提高政府工作的科学化水平。强化适应新常态、引领新常态的责任担当，始终保持奋发有为的精神状态，在推进产业升级、项目招引、城市建设等重点工作上，敢为人先，克难攻坚。加强学习型政府建设，深化务实性调查研究，着力培养想干事、会干事、干成事的干部队伍，为落实“十三五”规划和市委各项决策部署提供坚强保障。

　　创新管理转职能，推进治理现代化。加强法治政府建设，完善权力清单和责任清单制度，坚持依法科学民主决策，严格规范公正文明执法，充分发挥法律顾问作用。健全行政监督体系，主动接受人大的法律监督、工作监督和政协的民主监督，更加重视审计监督、社会监督和舆论监督。全面深化政务公开，畅通政府信息发布与公众互动交流的新渠道。转变经济工作方式，常态化开展“三服务”和中小企业服务周活动，减少直接干预企业微观经济行为，保障各类企业公平参与市场竞争。

　　创新机制提效能，树立政府新形象。以建市20周年为契机，大力弘扬建市之初的创业精神和“四个长期”、“三个不相信”精神。坚持民生工作例会制度，继续用好“三个平台”，深入开展“三解三促”，切实解决群众反映强烈的突出矛盾和问题。严格抓落实的工作机制，对重大项目和重要实事加大督查推进力度，加强跟踪问效、监察问责。健全改进作风长效机制，认真贯彻中央八项规定和省、市委十项规定精神，突出抓早抓小，持之以恒纠正“四风”。落实党风廉政建设责任制，坚持用制度管权、管事、管人，始终把纪律挺在前面，严肃查处各类违纪违法案件，树立为民务实清廉形象。

　　各位代表，发展蓝图已经绘就，宏伟目标催人奋进。让我们更加紧密地团结在以习近平同志为总书记的党中央周围，在中共泰州市委的领导下，解放思想、务实苦干，凝心聚力、开拓进取，为加快建设“四个名城”、全面建成小康社会、谱写好“迈上新台阶，建设新江苏”的泰州篇章而不懈奋斗！
